# Perioperative morbidity in total knee arthroplasty

**DOI:** 10.11604/pamj.2019.33.233.19095

**Published:** 2019-07-19

**Authors:** Khairreddine Raddaoui, Wafa Khedhri, Karima Zoghlami, Mohamed Radhouani, Emna Trigui, Olfa Kaabachi

**Affiliations:** 1 Department Of Critical Care and Anaesthesiology, Kassab Orthopaedic Institute, Faculty Of Medicine Of Tunis, Tunis El Manar University, Tunisia

**Keywords:** Total knee arthroplasty, perioperative, complications, risk factors, outcome

## Abstract

**Introduction:**

As the life expectancy and weight of patients are increasing, more old and obese patients are undergoing total knee arthroplasty (TKA). TKA may lead to several perioperative complications. These include anesthesia-related risks, exacerbation of comorbid medical issues and complications of surgical procedure. We have no studies reporting medical complications following TKA among our population. This study aimed to evaluate perioperative complications of TKA and to identify the related risk factors.

**Methods:**

It was a monocentric retrospective including 410 observations in the local TKA registry. Data of patients operated for primary unilateral TKA during the period from January 2014 to December 2017 were reviewed. All patients had standardized protocols of anesthesia and post operative care for three days following surgery. Multivariate logistic regression was used to identify the predicting factors for complications.

**Results:**

Incidence of perioperative complications was 37.1%. The most frequent were per operative hypotension (14.1%) and postoperative desaturation (21.7%, including pulmonary embolism in 2.4%). Multivariate logistic regression analysis identified: age ≥ 65 years (OR=1.9; p=0.006), respiratory diseases (OR=1.8; p=0.042) and general anesthesia (OR=2.8; p=0.009) as significant risk factors for any complications. Loss of autonomy (OR=4.8; p <0.001) and general anesthesia (OR=2.6; p=0.03) were significant risk factors for hypotension. Age ≥ 65 years (OR=2.6;p<0.001), female gender (OR=4.3;p=0.006) and respiratory diseases(OR=1.9;p=0.02) were associated with postoperative desaturation.

**Conclusion:**

This study highlighted hemodynamic and respiratory complications as the most common early complications in TKA. Age ≥ 65years, general anesthesia and respiratory diseases were significant risk factors.

## Introduction

Total knee arthroplasty (TKA) may be needed to alleviate pain and restore the ability of daily living's activities in advanced stages of Osteoarthritis. In the United States, logistic regression modelling suggests the incidence of TKA is expected to increase 69% by 2050 compared to 2012, from 429 procedures/100,000 in 2012 to 725 procedures/100,000 in 2050 [1]. In Tunisia although TKA has become a rife procedure because of the ageing of our population and the rising prevalence of overweight especially among women [2]. But, the prevalence of TKA is not known due to the lack of registries in orthopaedics. As we mentioned its drastic progression, TKA may lead to several perioperative complications. Adverse outcomes include acute myocardial infarction [3, 4], venous thromboembolism [5], pulmonary complications [6], sepsis [7, 8] and non myocardial cardiac complications [3, 7, 9]. Few well-designed studies, have examined all complications following TKA and with adjustment for potential confounding variables, have focused on risk factors such as age, sex, weight, specific medical co morbidities and surgical parameters and their effect on postoperative mortality, morbidity, and length of hospital stay following TKA. The aim of our study was to elucidate short-term complications occurring intra operatively and during the three days following primary TKA. The second aim was to identify risk factors for such complications.

## Methods

**Type of study:** it was a monocentric descriptive, analytic and retrospective study conducted in a referral center of orthopedic surgery (Department of critical care and anaesthesiology of Kassab Orthopaedic Institute, Tunisia) during the period from January 2014 to December 2017. We had enrolled all the patients of this period since we had standardized protocols of anesthesia and post operative care for at least three days following surgery. TKA register is the first database in Tunisia which is recording all the medical details of total knee replacement done in Kassab Orthopaedic Institute. Ethical approval was granted from local ethics committee, number CE 001/2014.

**Patients:** we included all the patients undergoing TKA for osteoarthritis (idiopathic or posttraumatic), inflammatory arthritis (rheumatoid, psoriatic, etc), or congenital deformities. We didn't included patients operated for simultaneous bilateral TKA, revision of TKA, primary unicompartemental knee arthroplasty (UKA) and revision of UKA, mmultiple joint replacements within the same admission and bone tumor. We included 410 observations in this database.

**Study procedures:** all patients had preoperative assessment three months maximum before surgery. Patients had standard monitoring during surgery. Spinal anesthesia was performed unless contraindication after loading with 500 mL saline. Single shot spinal anesthesia using 12 mg of hyperbaric bupivacaine 0.5% and 5 micrograms of sufentanyl is performed in patients aged less than 65 years, otherwise we used continuous spinal anesthesia using boluses of 2.5 mg isobaric bupivacaine 0.5% and 2.5 micrograms of sufentanyl. General anesthesia was indicated in case of no indication of spinal anesthesie or its failure. Cefuroxime was employed as antibiotic prophylaxis. All patients received four dose of 10 mg/kg tranexamic acid within surgery at and H3 and H6 postoperatively. All patients were operated under pneumatic tourniquet. Routine analgesia protocol used: ultrasound guided continuous perineural block (femoral nerve block with or without sciatic nerve block, or saphenous nerve block) with either 0.125% bupivacaine or 0.2% ropivacaine for 48H and multimodal systemic analgesia with: 3-4g paracetamol/day, non steroidal anti inflammatory for 5 days and PCA (Patient Controlled Analgesia) morphine for 48h. Postoperative rehabilitation started on day one. Thrombophylaxis was beginning at H6 postoperatively with enoxaparin: 0.4ml if BMI (body mass index) <40kg/m^2^ or 0.6 ml if BMI ≥40kg/m^2^. Transfusion was indicated if severe anemia and hemodynamic instability. We measured creatinine serum, hemoglobin and tropnin on day one. We also performed postoperative electrocardiogram. Patients were discharged to orthopedic ward at day 3 postoperatively if they were able to walk and had no short term complications.

**Collected data:** patients characteristics available in the Kassab TKA register database were divided into:

**Demographic characteristics:** age, gender, BMI, autonomy physical status and metabolic equivalent of task (MET), ASA (American Society of Anesthesiology) classification, cardiovascular and respiratory co morbidities, diabetes mellitus, chronic renal failure defined by a clearance of creatinine less than 60 milliliters/minute and preoperative hemoglobin.

**Intra operative variables:** general or spinal anesthesia, tourniquet pressure and duration, intra operative hypotension, arrhythmia, bradycardia, tachycardia and desaturation.

**Postoperative variables:** temperature, blood pressure, heart rate, respiratory rate, peripheral oxygen saturation, urine output, postoperative bleeding, haemoglobin level, creatinine serum level, blood transfusion, blood gas, troponin level. We performed also electrocardiogramm, Chest x-ray, duplex ultrasonography, cerebral scan, thoracic computed tomography scan if needed. Length of stay in the postoperative recovery unit was mentioned.

**Perioperative complications were divided into:** *hemodynamic events:* hypotension defined by decrease of ≥20% of preoperative baseline values. Tachycardia defined by increase of ≥20% of preoperative baseline values. Myocardial infarction as defined by European societies of cardiology and anesthesiology. *Respiratory events:* desaturation was defined as a pulse oxymeter value lower than 94% or a >5% decrease of baseline preoperative value. Early desaturation occurring during the first 24 hours and spontaneously resolving under oxygen therapy within the next 24 hours was considered as bone cement implantation syndrome (BCIS). Desaturation occurring later than the first 24 hours or lasting more than 24 hours and improving with positioning and respiratory physiotherapy was considered as atelectasis and mild lung collapse. Desaturation persisting beyond 48 hours despite positioning and respiratory physiotherapy measures justified thoracic angio tomodensitometry looking for pulmonary embolism (PE). *Thrombotic events:* deep venous thrombosis (DVT) or pulmonary embolism. *Neurological events:* stroke or cognitive disorders was established by a neurological deficit or cerebral CT scan. *Renal events:* acute kidney injury was defined by RIFFLE criteria: ≥1.5 folds increase in creatinine concentration and/or urinary output <0.5 ml/g/h for ≥6hours.

**Statistical analysis:** all statistical analysis was performed using SPSS version 22 on windows (IBM Corp, Armonk, NY, USA). Descriptive statistics were obtained on all variables used within the study. Missing values were not excluded for the purpose of this study. Univariate logistic regression and the chi-square statistic were used to determine the effect of individual risk factors on mortality, the development of complications as well as respiratory and hemodynamic complications or cardiac complications and the length of stay. The primary patient predictors included age (categorized ≥ 65 years old vs <65 years old), gender, BMI (categorized as ≥30kg/m^2^vs <30kg/m^2^), ASA classification (1 and 2 compared with 3), MET (categorized <4 vs ≥4), autonomy physical status (categorized mild and moderate versus severe), habits, co morbidities, anesthetic procedures and transfusion. Multivariate logistic regression was conducted on risk factors with a p value <0.15 in the initial univariate analysis. Significant risk predictor factors were determined to be those that maintained p values of <0.05 with the OR and 95% CI exclusive of 1.0 following multivariate analysis.

## Results

The mean age of our population was 65.8±7.8 years. The majority of the patients were female in 85.1% with a BMI of ≥30 kg/m^2^ in 74.6% (Table 1).The most common medical co morbidities were hypertension (66.1%), diabetes (22.2%) and respiratory diseases (20.5%) (Table 1). Patients had moderate to severe autonomy physical status in 92% of cases. Only 34 patients (8.3%) had general anesthesia. The mean duration of tourniquet (assessed rather than operative time) was 124.9±22.3 minutes. Length of stay in postoperative care unit was prolonged beyond three days in 36.7% of patients. Almost 37.1% of our patients presented one or more perioperative short term complication. The most frequent complications were intra operative hypotension (14.1%) and postoperative desaturation (21.7% including: 9.8% atelectasis, bone cement implantation syndrome in 9.3% and pulmonary embolism in 2.4%) (Table 2). Postoperative bleeding was up to 500 ml in 47.3% of cases and only 15.4% of patient required blood transfusion. There was no in-hospital mortality in our serie. On univariate analysis, we found that significant risk factors were for: any complication: Age>65 years (OR 2.1; 95%CI, 1.4 to 3.2); severe autonomy physical status (OR 1.7;95%CI, 1.04 to 2.7); MET <4 (OR 1.84; 95% CI, 1.09 to 3.11) and respiratory diseases (OR 1.8; 95% C, 1.1 to 3.0); Intra operative hypotension: severe autonomy physical status (OR 4.9; 95% CI, 2.6 to 9.2) and MET <4 (OR 4.9; 95%CI, 2.6 to 9.4); Acute renal failure: age >65years rate (OR 2.6; 95%CI, 1.2 to 5.5); respiratory diseases (OR 4.0; 95%CI, 1.1 to 14.3) and chronic kidney diseases (OR 11.1; 95%CI, 2.6 to 46.6). Risk factors in univariate analysis for desaturation were repported in Table 3 and for length of stay were reported in (Table 4). Multivariate analysis ascertained that a patient older than 65 years was the most important risk factor for the development of one or more perioperative complications (Table 5).

**Table 1:** patient demographic characteristics

Characteristics		Value
**Age (N=410)**	**Mean and standard deviation (year)**	**65.8± 7.8**
	**Age groups N (%)**	
	59yr	61 (14.9%)
	60-69yr	226 (55.1%)
	70-79yr	112 (27.3%)
	≥80yr	11 (2.7%)
**Gender (N=410) N (%)**	Male	61 (4.9%)
	Female	349 (85.1%)
**BMI (N=382)**	Mean (kg/m²)	33.8 ± 6.0
	BMI groups N (%)	
	≤24.9kg/m²	26 (6.8%)
	25-29.9kg/m²	71 (18.6%)
	30-34.9kg/m²	125 (32.7%)
	35-39.9kg/m²	97 (25.4%)
	≥40kg/m²	63 (16.5%)
**Smoking N (%)**		
Hypertension N (%)		271 (66.1%)
Diabetes N (%)		91 (22.2%)
Respiretory diseases N (%)		84 (20.5%)
Chronic kidney disease N (%)		37 (9%)
Arrhythmia N (%)		24 (5.9%)
Pulmonary hypertension N (%)		30 (7.3%)
Heart failure N (%)		10 (2.4%)
Valvulopathies N (%)		12 (22.6%)
Dysthroidism N (%)		24 (5.9%)
Anemia N (%)		85 (20.7%)
ASA Classification (N=410) N (%)	ASA1	74 (18.0%)
	ASA2	231 (56.0%)
	ASA3	105 (25.6%)
Metabolic equivalent (N=389) N (%)	≥4	319 (82.0%)
	<4	70 (18.0%)
Autonomy* (N=385) N (%) 0 or 1: mild		8 (2%)
	2: moderate	291 (71%)
	3 or 4: severe	86 (21%)

**Table 2:** total number and percentage of complications

Complications	N (%)
Any complication	152 (37.1)
Intraoperative hemodynamic and cardiac complications	58 (14.1)
Hypotension	50 (12.2)
Tachycardia	7 (1.7)
Arrhythmia	1
Postoperative hemodynamic and cardiac complications	33 (8)
Hypotension	19 (4.6)
Tachycardia	10 (2.4)
Arrhythmia	4 (1)
Myocardial infarction	0
Postoperative desaturation	89 (21.7)
Bone cement implantation syndrome	38 (9.3)
Pulmonary Embolism	10 (2.4)
Presumed Atelectasis	40 (9.8)
Pulmonary oedema	1
Respiratory arrest (opioid induced)	1
Venous thrombosis	1
Acute renal failure	8 (2)
Neurological issues(stroke, cognitive disorders)	2
Infection	0

**Table 3:** influence of risk factors related to general characteristics on respiratory complications (Univariate and chi-square analysis)

Risk Factors		Postoperative desaturation			Bone cement implantation syndrome			Atelectasis			Pulmonary embolism		
		%	P	OR	%	p	OR	%	p	OR	%	P	OR
**Age**	>65yr (N=213)	30	<0.001	2.9	12.2	0.03	2.1	12.2	0.01	2.6	3.8	0.1	-
	≤65yr (N=197)	12.7			6.1			5.1			1		
**Gender**	Female(N=349)	24.1	0.006	3.5	10	0.2	-	4.9	0.2	-	2.9	0.18	-
	Male (N=61)	8.2			4.9			9.5			0		
**Diabetes**	Yes (N=91)	30.8	0.017	1.88	9.9	0.8	-	9.9	0.6		6.6	0.01	5.5
	No (N=319)	19.1			9.1			8.5			1.3		
**Respiratory Diseases**	Yes (N=84)	35.7	<0.001	2.5	13.1	0.1	-	13.1	0.1		6	0.03	4.0
	No (N=326)	18.1			8.3			7.7			1.5		

**Table 4:** influence of risk factors related to complications on length of stay (univariate and chi-square analysis)

Risk Factors		Length of stay of > 3 days %	*p*	OR
**Any complication**	Yes (N=152)	51.7	<0.001	2.7 (1.8 to 4.1)
	No (N=258)	27.9		
**Postoperative desaturation**	Yes (N=88)	62.5	<0.001	3.9 (2.4 to 6.4)
	No (N=321)	29.6		
**Bone Cement implantation syndrome**	Yes (N=38)	52.6	0.032	2.0 (1.05 to 4.03)
	No (N=371)	35		
**Atelectasis**	Yes (N=35)	60	0.003	2.8 (1.4 to 5.7)
	No (N=374)	34.5		
**Pulmonay embolism**	Yes (N=10)	100	<0.001	2.8 (2.4 to 3.2)
	No (N=399)	35.1		

**Table 5:** multivariate analysis of independent risk factors for intra operative, postoperative complications and length of stay

Risk factors	*p* value of crude OR	Crude OR (95% CI)	*p* value of adjusted OR	Adjusted OR
**Any complication**				
Age-65years	<0.001	2.1 (1.4 to 3.2)	0.006	1.9 (1.2 to 3.0)
Respiratory diseases	0.013	1.8 (1.1 to 3.0)	0.042	1.8 (1.1 to 2.9)
Anesthetic procedure	0.0.6	1.9 (0.9 to 3.8)	0.009	2.8 (1.2 to 6.1)
Intraoperative hypotension				
Autonomy (Physical status)	<0.001	4.9 (2.6 to 9.2)	<0.001	4.8 (2.5 to 9.2)
Anesthetic procedure	0.01	2.8 (1.25 to 6.6)	0.03	2.6 (1.0 to 6.3)
**Postoperative desaturation**				
Age-65 years	<0.001	2.9 (1.7 to 4.2)	0.001	2.6 (1.5 to 4.5)
Gender(female)	0.006	3.5 (1.3 to 9.17)	0.006	4.3 (1.5 to 12.6)
Respiratory disease	<0.001	2.5 (1.2 to 4.0)	0.026	1.9 (1.08 to 3.4)
**Bone cement implantation syndrome**				
Age 65 years	0.03	2.1 (1.0 to 4.3)	0.02	2.9 (1.1 to 7.3)
Atelectasis				
Age-65 years	0.01	2.6 (1.2 to 5.5)	0.01	2.4 (1.1 to 5.3)
Pulmonary embolism				
Age -65years	0.07	3.8 (0.7 to 18.4)	0.001	2.7 (1.5 to 4.8)
**Acute renal failure**				
Chronic renal failure	0.003	11.1 (2.6 to 46.6)	0.002	14.4 (2.7 to 76.3)
Intraoperative hypotension	<0.001	13.2 (3.0 to 57)	0.008	8.8 (1.7 to 44.9)
Postoperative hypotension	<0.001	14.4 (3.1 to 65.9)	0.017	9.1 (1.4 to 56.4)
**Length of stay**				
Postoperative desaturation	<0.001	2.7 (1.8 to 4.1)	<0.001	5.9 (2.9 to 11.8)

## Discussion

In our study we wanted to expose intra operative and early three days medical post operative complications while patients were admitted in postoperative recovery unit and to assess their predictive risk factors. We didn't report any death during the study period. In our study, 37.1% of the patients developed one or more complication. Pulmonary complications were the most frequent postoperative events occurring in 21.7% of patients. Most of them were considered as moderate postoperative events (brief desaturation in 9.3% and atelectasis in 9.8%). However we reported a high rate of pulmonary embolism in 2.4%. Patients older than 65 years were at risk for developing any complication. This study is the first to evaluate medical complications during the surgery and within three days following TKA in Tunisia. Data of TKA were obtained from local registry of Kassab which is unique in Tunisia. One important fact to consider is that this study was performed in a single institution where standardized protocols are in place and patients receive a thorough preoperative medical evaluation and are followed by intensivist postoperatively. However, several limitations of this study should be noted. First, our study is a single-center and have relied on local register. Second, our population included a small number of patients compared with large published series. Finally, the short follow-up of patients (three postoperative days) didn't allow detection of complication that occurred after discharge. Concerning the same topic the dataset did not provide information regarding long-term outcomes and other clinical outcomes such as peripheral nerve injury. TKA has been associated with serious intraoperative and postoperative complications and occasionally even with death [10]. So it is imperative for clinicians to be aware of factors that are associated with this unfortunate outcome. In our study, there was no case of death during hospital stay. In a recent systematic review involving thirty-seven studies with mortality data from 15 different countries following over 1.75 million TKA, the pooled Poisson-normal random-effects meta-analysis estimates of 30 and 90-day mortality were 0.20% (95% CI, 0.17% to 0.24%) and 0.39% (95% CI, 0.32% to 0.49%) [3]. It was reported that cardiovascular causes, particularly myocardial infarction, were cited as the most common cause of death in several studies [4, 5]. Diabetes, increasing age and male gender were also reported as independents risks factors for mortality [4, 6-8]. Our result may be explained by the short follow-up limited to three days In these study, 37.1% of the patients developed one or more complication. Rate of complications in our study was high comparing with others studies. The most frequent were intraoperative hypotension (14.1%) and postoperative desaturation (21.7% including 9.8% atelectasis, bone cement implantation syndrome in 9.3% and pulmonary embolism in 2.4%). In a retrospective study involving 2033 patients, Huddleston *et al* showed [9], that 132 (6.5%) patients experienced at least one adverse event during their hospitalization. Another large series reported 1057 complications among 851 (5.55%) of the patients [4]. The major systemic events were cardiovascular in 46.6%, with pulmonary embolism being the most prevalent major systemic complication (noted in 0.78% of the patients).There was an increase in the incidence of major complications: pulmonary embolism, sepsis, myocardial infarction cardiac complications and pneumonia as reported by Kirksey *et al* [11]. With the shorter follow-up in our study (3 days VS 30 to 90 days), one would expect to see lower rather than higher rate of complications. One explanation for this may be related that we were interested also in intraoperative complications. We also focused in desaturation, that occurred in 21.7%, but, was really associated with relevant clinical event in only 9.8% with atelectasis and 2.4% with pulmonary embolism.

In our study, multivariate analyses demonstrated that a patient older than 65 years (OR=1.9; 95% CI, 1.2 to 3.0), pre-existing respiratory diseases (OR=1.8; 95% CI, 1.1 to 2.9) and general anesthesia (OR=2.8; 95%, CI, 1.2 to 6.1) were the most important risk factors for developing of any complication. Age was an important predictor of early complications. This association has been described in numerous studies [4, 12, 13]. Furthermore, Higuera CA *et al* showed that patients were approximately 40% more likely to have any complication per each subsquent 10 years of age [13]. In our study, patients with respiratory disease had elevated risks for postoperative complications. Pre-existing chronic obstructive pulmonary disease was risk factors for in-hospital complications [11, 14]. Such patients were at significantly increased risk of any complication including increased mortality, pneumonia, re intubation, use of a mechanical ventilator for >48 hours, cardiac arrest, progressive renal insufficiency, deep infection, return to operating room and a readmission within 30 days postoperatively [15]. Our results demonstrated that patients who were managed with general anaesthesia had significant increase in the risk of complications. Such result was found in many studies in the literature [16-18]. Similarly to our data, the BMI was not a good predictor of adverse outcomes or complications [13]. BMI ≥40 kg/ m^2^ was the most important risk factors for developing of any complication in one study [4]. In our study we didn't reported any case of myocardial infarction. In contrast, many studies showed that cardiac diseases were associated with the risk of a postoperative nonsurgical complication [9, 13, 14, 19]. Few patients in our study suffered from either chronic heart failure or coronary artery diseases. Postoperative desaturation was the first highest incidence complication and the major finding that caught our attention while settling our TKA register, as we already mentioned. Most of the cases of postoperative desaturation (9.3%) happened early in postoperative period, lasted few hours and were classified as BCIS. In fact, its incidence following TKA is not clearly identified in literature since there is no agreed definition of this syndrome and thus it may be misdiagnosed. Donaldson *et al*, recently proposed a severity classification of BCIS that we followed in our study [20]. In our study, an age over 65 years with poor pre-existing physical reserve were found as risk factor for developing BCIS. Numerous patient-related risk factors have been implicated in the genesis of BCIS including old age, poor pre existing physical reserve, impaired cardiopulmonary function, pre existing pulmonary hypertension, osteoporosis, bony metastases, and concomitant hip fractures, particularly pathological or intertrochanteric fractures [20].

BCIS incidence seemed to be high in our patients. It may be explained by the poor pre-existing physical reserve and chronic respiratory diseases. In our study the rate of atelectasis among our patients was 9.8%. Only, old age was a risk factor for atelectasis. Austin *et al*. [21] found in a prospective study including 1058 TKA that among postoperative hypoxemia, atelectasis was the most common etiology with a rate of 33%. The main risk factors for developing atelectasis in the surgical patient include: general anaesthesia, the site of surgery, high BMI, blood transfusion and diabetes mellitus [21]. In our study, the rate of atelectasis seems to be high as regional anesthesia was performed in most patients. It can be explained by: increasing age, poor functional status, prolonged bed rest before surgery, patients with underlying chronic lung diseases, long duration of surgery (the mean of tourniquet time is 125 minutes), obesity (Mean weight is about 35 kg/m^2^), the mean dose of daily morphine consumption was 20 to 24 mg and late mobilisation after 24 hours of surgery. According to our hospital registry data, the overall incidence of pulmonary embolism was 2.3% which is a high rate in comparison with asian and western population. In a taiwanese retrospective study carried out between 2007 and 2010, five patients (0.28%) developed a symptomatic pulmonary embolism in a series of 1768 patients [22]. According to a study of 222,684 TKA in USA between 1990 and 2004, the incidence of PE was 0.41% in primary TKA [23]. Kirksey *et al*. showed a significant trend of increasing rates of the diagnosis of PE after TKA between 1998 and 2008 [11]. Adoption of computer tomography pulmonary angiography in the detection of PE since 1998 has been shown to have led to an overall increase in the number of events [24]. In our study, age was the only risk factor for developing pulmonary embolism after TKA (OR=2.7; 95%CI, 1.5 to 4.8). In A large database, most risk factors for embolism events were: cerebrovascular disease, obesity, older age, female sex and bilateral surgery, surgery time > 2 hours [25]. In our study, we didn't find increased risk of PE with increased BMI which was similar to other studies [26]. Pulmonary embolism at a rate of 2.7% of patients exceeds reported incidence in previous studies that is usually around 1%. We think that, besides the above mentioned statistically significant factors, there are other potential causes. Eventually, our thrombophylaxis strategy based on 40 mg of enoxaparine (60 mg for morbidly obese patient) may not be best suitable for this particular embolism high risk surgery comparative to the Food and Drug Administration (FDA) and other American colleges guidelines of 30 mg twice a day for TKA (40 mg twice daily if BMI ≥40 kg/m^2^). Actually, Sadeghi *et al*. in a case-control study including 593 TKA records abstracted by 15 participating teaching hospitals concluded that suboptimal pharmacologic prophylaxis was significantly associated with venous thromboembolism (PE and DVE) after TKA [27]. In our study, we looked for intra operative events, especially intra operative hypotension that was frequent in our patients and it was a risk factor of developing renal failure postoperatively. Mortazavi *et al*. suggest that intra operative arrhythmia and blood pressure fluctuations are associated with increased risk of perioperative stroke [28]. We found that a poor status of autonomy and general anesthesia were a risk factors of developing intraoperative hypotension. Nwachukwu *et al* reported that intra operative hypertension and obesity increase the likelihood of poor blood pressure control during TKA [29]

## Conclusion

Analysis of our database on TKA demonstrated a high incidence of perioperative morbidity, particularly respiratory complications. The main risk factors were age over 65 years and high co morbidity burden especially respiratory diseases. The identification of patients with such risk factors is paramount to doctors for better preoperative medical optimization and strict postoperative care.

### What is known about this topic

- Joint arthroplasty is comimng a surgical procedure as population is aging. Recent literature focused on increasing rate of medical complications following knee artroplasty. Several risk factors had been identified. Identifying such population could improve perioperatoire care and reduce postoperative morbidity.

### What this study adds

- Our study is the first one in our country and may be also in non developed world. We demonstrated that postoperative respiratory complications were frequent, unlike most of literature. Most of such complications were mild with on in-hospital mortality

## Competing interests

The authors declare no competing interest.
